# Implementation fidelity of a nurse-led RCT-tested complex intervention, care coordination for health promotion and activities in Parkinson’s disease (CHAPS) in meeting challenges in care management

**DOI:** 10.1186/s12883-021-02481-5

**Published:** 2022-01-24

**Authors:** Karen I. Connor, Hilary C. Siebens, Brian S. Mittman, David A. Ganz, Frances Barry, Donna K. McNeese-Smith, Eric M. Cheng, Barbara G. Vickrey

**Affiliations:** 1 Veterans Affairs Parkinson’s Disease Research, Education and Clinical Center, Los Angeles, CA USA; 2 UCLA David Geffen School of Medicine, Los Angeles, CA USA; 3 Novato, CA 94945 USA; 4 Siebens Patient Care Communications LLC, Seal Beach, CA USA; 5 Kaiser Permanente Department of Research and Evaluation, Pasadena, CA USA; 6 Veterans Affairs Geriatric Research, Education and Clinical Center and Center for the Study of Healthcare Innovation, Implementation and Policy, Los Angeles, CA USA; 7 UCLA School of Nursing, Los Angeles, CA USA; 8 Icahn School of Medicine at Mount Sinai, New York, NY USA

**Keywords:** Parkinson’s disease, Patient care management, Nursing process, Health communication, Case manager, Implementation fidelity, Dissemination

## Abstract

**Background:**

Parkinson’s disease (PD) complexity poses challenges for individuals with Parkinson’s, providers, and researchers. A recent multisite randomized trial of a proactive, telephone-based, nurse-led care management intervention - Care Coordination for Health Promotion and Activities in Parkinson’s Disease (CHAPS) - demonstrated improved PD care quality. Implementation details and supportive stakeholder feedback were subsequently published. To inform decisions on dissemination, CHAPS Model components require evaluations of their fidelity to the Chronic Care Model and to their implementation. Additionally, assessment is needed on whether CHAPS addresses care challenges cited in recent literature.

**Methods:**

These analyses are based on data from a subset of 140 intervention arm participants and other CHAPS data. To examine CHAPS Model fidelity, we identified CHAPS components corresponding to the Chronic Care Model’s six essential elements. To assess implementation fidelity of these components, we examined data corresponding to Hasson’s modified implementation fidelity framework. Finally, we identified challenges cited in current Parkinson’s care management literature, grouped these into themes using open card sorting techniques, and examined CHAPS data for evidence that CHAPS met these challenges.

**Results:**

All Chronic Care Model essential elements were addressed by 17 CHAPS components, thus achieving CHAPS Model fidelity. CHAPS implementation fidelity was demonstrated by adherence to content, frequency, and duration with partial fidelity to telephone encounter frequency. We identified potential fidelity moderators for all six of Hasson’s moderator types. Through card sorting, four Parkinson’s care management challenge themes emerged: unmet needs and suggestions for providers (by patient and/or care partner), patient characteristics needing consideration, and standardizing models for Parkinson’s care management. CHAPS activities and stakeholder perceptions addressed all these themes.

**Conclusions:**

CHAPS, a supportive nurse-led proactive Parkinson’s care management program, improved care quality and is designed to be reproducible and supportive to clinicians. Findings indicated CHAPS Model fidelity occurred to the Chronic Care Model and fidelity to implementation of the CHAPS components was demonstrated. Current Parkinson’s care management challenges were met through CHAPS activities. Thus, dissemination of CHAPS merits consideration by those responsible for implementing changes in clinical practice and reaching people in need.

**Trial registration:**

ClinicalTrials.gov as NCT01532986, registered on January 13, 2012.

## Background

The complexity of health care delivery in Parkinson’s disease (PD)^1^ poses challenges for individuals with Parkinson’s, providers, and researchers; thus, health service and implementation researchers are examining new Parkinson’s care models to address gaps in care and delivery system problems [1, 2]. These include multi-component interventions involving many disciplines addressing patients’ needs while supporting providers [3–13]. For example, prevention and treatment of fall risks/falls is a priority concern for patients with enduring (chronic) conditions like Parkinson’s [14], requiring communication, collaboration, and coordination among patients, care partners, and health care team members. Specific responsibilities require: (1) identifying fall risks/falls as a problem (by physicians and nurses), (2) considering medication changes (by physicians), (3) assessing and managing unsafe behaviors (by mental health and care team), (4) assessing ways to make daily activities safer (by patients, care partners, nurses, physical and occupational therapists), and (5) providing care partner education and support (by nurses, occupational and physical therapists, and social services).

Our new Parkinson’s care model is the proactive nurse-led telephone-based care management intervention, Care Coordination for Health Promotion and Activities in Parkinson’s Disease (CHAPS). CHAPS evolved from prior health services research on a dementia care management program, Alzheimer’s Disease Coordinated Care for San Diego Seniors (ACCESS), that was based on the Chronic Care Model [15], a widely used framework with strategies to facilitate productive, patient-centered communications and interactions for providing high quality chronic disease care. The Chronic Care Model is comprised of six essential elements: (1) health system resources and policies, (2) community resources and policies, (3) delivery system redesign, (4) decision support, (5) clinical information systems, and (6) self-management support. ACCESS achieved higher care quality on 21 of 23 (*p* < 0.013 for all) dementia guideline recommendations [16]. For CHAPS, health services researchers designed the CHAPS intervention with input from direct-care nurses and providers to meet a set of 38 PD quality indicators.

To choose the set of indicators, the researchers pulled 106 PD indicators and guidelines from multiple sources: Assessing Care of Vulnerable Elders (ACOVE), the American Academy of Neurology, National Institute for Clinical Excellence, European Federation of Neurological Societies, and the Parkinson’s Disease Research, Education and Clinical Centers (PADRECC) Quality Indicator Project [EMC, KIC, BGV, PD Rating Booklet 2009, unpublished]. Then a Task Force of Parkinson’s disease specialists, nurse educators, representatives from community organizations committed to Parkinson’s, and a nurse working in a movement disorder clinic rated these 106 indicators and guidelines on validity and their room for improvement. The result was a set of 38 indicators used to guide the design of the CHAPS intervention [7]. CHAPS was then implemented and compared to usual care in a randomized controlled trial, by measuring adherence to 18 indicators, of the 38, that were likely sensitive to care management.

### Parent study

In brief, we conducted the CHAPS trial between 2012 and 2017 at five Veterans Health Administration medical centers in the Southwest United States. Patient/participants were community-dwelling men and women who had been in the United States military (i.e., veterans) in the care of one of the five sites and were the unit of randomization. Potential participants had at least 2 International Classification of Disease − 9 (ICD 9) diagnostic codes over 12 months prior to the study and were not already enrolled in an existing care management program (e.g., congestive heart failure, diabetes). Of note, these study participants had more disability compared to another community-based population (Health Utilities Index 3 mean 0.46 versus 0.61) [17].

The intervention commenced with the CHAPS Initial Assessment tool administered by the CHAPS nurse care manager. The Assessment’s embedded algorithms identified 31 standard problems/topics. A 3-ringed binder, the Siebens Health Care Notebook (Notebook), a self-care, communication, and navigational tool, was personalized by the CHAPS nurse care manager and mailed to each participant [18]. This Notebook was a single location to keep an individual’s health care information. This included (1) a sheet with the nurse care manager’s photograph and contact information (telephone number and mailing address), (2) an updated medication list, (3) personalized next steps in “My Action Plan”, (4) tailored education sheets, and (5) a copy of the individual’s CHAPS Assessment to show other providers. Nurse care managers scheduled a specific time to teach Notebook use and included care partners at participants’ request. Nurse care managers also provided routine follow-up calls to coach participants on problems/topics identified - guided by problem/topic-specific intervention protocols - at 6 months, at annual reassessment, and at 18 months [7]. Interim follow-up calls occurred as needed. The Siebens Domain Management Model™ (I Medical/Surgical Issues, II Mental Status/Emotions/Coping, III Physical Function, and IV Living Environment) served as CHAPS’ person/patient-centered Organizing Framework to guide and document holistic care [14, 19].

Decision support occurred through the problem/topic-specific intervention protocols, huddles between the CHAPS nurse care managers and Parkinson’s disease specialists, and through meetings among the nurse care managers. These nurse care managers interacted with other health care professionals (e.g., primary care providers and medical specialists, social workers, speech language pathologists, physical and occupational therapists) through routine medical record documentation and warm hand-offs (e.g., in person, by telephone, by secure email, and/or co-signature requests of medical record notes).

The CHAPS intervention improved care quality through increased adherence to the 18 PD quality of care indicators [17] relative to usual care. We subsequently published details of the CHAPS implementation [14] and the overall positive feedback provided by stakeholders (CHAPS nurse care managers, Parkinson’s disease specialists, and patients/participants) [20].

The first goal of the study reported here is to evaluate the fidelity of the CHAPS Model to the Chronic Care Model as this establishes six essential model elements have, in fact, been applied in the new CHAPS intervention. The second goal is to evaluate fidelity to implementation of the CHAPS components. Examining implementation fidelity contributes towards understanding an intervention and whether its components were operating as intended. This then increases reproducibility, confidence in attributing outcomes to these components, and potential for dissemination [21–26]. Several published frameworks for measuring fidelity vary slightly in scope and detail [23] yet have common core concepts of adherence and potential moderators [21, 22]. Because CHAPS was designed in 2011, the third goal of this study was to identify recently published challenges in Parkinson’s care management from 2012 to 2020 and assess if these challenges were addressed in the CHAPS implementation.

## Methods

**Fig. 1:**
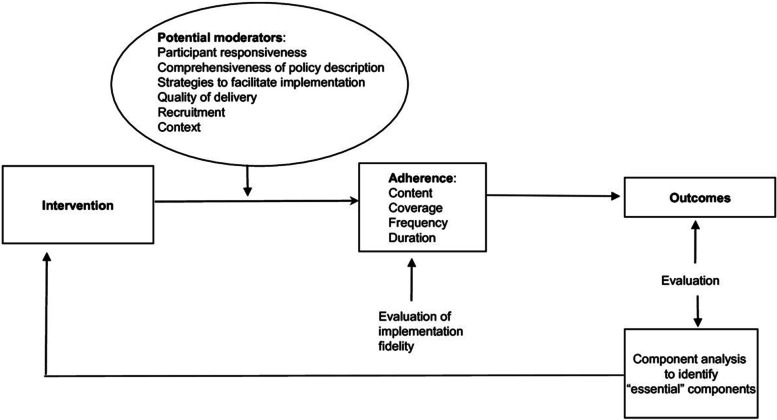
The modified conceptual framework for implementation fidelity (originally from Carroll et al). Legend: Fig. 1 is from Hasson H. Systematic evaluation of implementation fidelity of complex interventions in health and social care. BioMed Central Implementation Science 2010;5: 67, page 3

Aims of this descriptive study were to evaluate: (1) fidelity of the CHAPS Model to the Chronic Care Model’s six elements (i.e., were model components created for each element) [7, 15]; (2) fidelity of CHAPS Model implementation (i.e., were components used and protocols followed (adherence)); (3) evaluation of potential fidelity implementation moderators; and (4) if and how CHAPS addressed challenges in Parkinson’s care management cited recently in the literature. Implementation fidelity was based on the modified conceptual framework by Hasson [21] (Fig. 1).

### Setting and eligible participants

Participant recruitment in the CHAPS trial [17] was from a total of 452 candidates identified on chart review. The final number of trial participants was 328 (73% of the 452). A total of 166 were randomly assigned to usual care (control) and 162 to the CHAPS intervention [17]. The intervention subgroup for this fidelity study was 140 participants that received at minimum the CHAPS Initial Assessment administered by the CHAPS nurse care manager [14]. This subgroup had a mean age of 69.4 years (standard deviation (SD) 10.3 years), was 95% male, and had a mean Health Utilities Index 3 of 0.45 (SD 0.31) (range: − 0.36 (worst) to 1 (best)), a patient-reported outcome assessment of health-related quality of life [27]. These values did not differ significantly from the 22 intervention participants who did not receive CHAPS nurse care management for various reasons [14]. A total of 52% (*n* = 73/140) of the intervention subgroup received care from providers both within and outside the Veterans Affairs Medical Centers [14].

### Data

To assess fidelity to the Chronic Care Model [15], CHAPS’ theoretical model, two researchers (KIC, HCS) created a table of the Chronic Care Model’s six essential elements designed to encourage high quality care (Table 1). CHAPS Model components that corresponded to these elements were added to the table [7, 14]. To assess implementation fidelity to these CHAPS components, findings from a prior CHAPS publication [14] were reviewed for examples of adherence variables (Fig. 1) and entered in the table. Next, the six fidelity potential moderator types from Hasson’s model (Fig. 1) [21] were entered into another table (Table 2). Examples for these six types were identified from published CHAPS data [14, 17, 20], quantified where feasible, and added to the table. An additional data source was the Principal Investigator (nurse researcher, KIC) performing on site observation of care delivery and having discussions with CHAPS nurse care managers.

**Table 1:** Fidelity of the CHAPS Model and Its Implementation among *N* = 140 intervention study participants

Chronic Care Model Elements	Fidelity of the CHAPS Model Components to the Chronic Care Model [7]	Fidelity of CHAPS Implementation of CHAPS Model [14] to Hasson’s Model
Health System Resources and Policies		
	5 Veterans Affairs Healthcare Systems medical centers	Greater Los Angeles, Las Vegas, Loma Linda, Long Beach, San Diego (*Content*)
Community Resources and Policies		
	Local/regional/national organizations and services (local APDA, LA-CRC, NPF, and PRO; and other local Parkinson’s disease support groups)	Recommended use of community services (*n* = 78, 29.5% of all service referrals) (*Content*) (*Frequency*)
Delivery System Redesign		
	CHAPS nurse care managers	8 nurses hired and oriented to deliver CHAPS care management (*Content*)
	Parkinson’s disease specialist and CHAPS nurse care manager regularly scheduled huddles	CHAPS nurse care managers reported to principal investigator that huddles occurred monthly (*Content*) (*Frequency*)
	Telephone encounters with participants	*n* = 656 telephone encounter notes abstracted
		• *n* = 68 (prior to Initial CHAPS Assessments)
	➢ Initial CHAPS Assessments	• *n* = 140 (Initial CHAPS Assessments)
	➢ Follow-up calls	• *n* = 317 (~ 3 follow-ups per participant) ^a^
	➢ 6-month reassessments	• *n* = 67 (6-month reassessment, for 50% of eligible candidates
	➢ Annual reassessments	• *n* = 29 (Annual reassessment, for 23% of eligible candidates)
		• *n* = 35 (2nd 6-month reassessment at 18-months for 29% of eligible candidates) (*Frequency – partial fidelity*) (*Duration*)
	Siebens Domain Management Model™ (SDMM™) ^b^ as the organizing framework	Domain headings used in documentation of 4870 (97.7%) of CHAPS problems/topics. These distributed over the 4 domains: • Domain I 38.8% • Domain II 27.5% • Domain III 14.3% • Domain IV 19.4% (*Content*) (*Frequency*)
	Siebens Health Care Notebook [18]	• Personalized self-care tool sent to each participant (*n* = 140) ^c^ • Notebook discussed with participants n = 108 (77.1%) (*Content*) (*Frequency*)
Decision Support		
	31 standard CHAPS problems/topics identified through algorithms	Problems/topic types were documented (*n* = 4938) (*Content*) (*Frequency*)
	CHAPS problem/topic intervention protocols with care recommendations ^d^	CHAPS nurse care manager activities (*n* = 4012) (27 types) (*Content) (Frequency)*
	CHAPS nurse care manager meetings	Meetings twice a month for mutual support and clinical problem-solving (*Content*) *(Frequency)*
	Decision support provided by Parkinson’s disease specialists directly to CHAPS nurse care managers through huddles	CHAPS nurse care managers reported to principal investigator huddles occurred monthly (*Content*) (*Frequency*)
Clinical Information Systems		
	Computerized Patient Record System - CPRS (Veterans Affairs electronic medical record for inpatient and outpatient services)	CHAPS nurse care manager notes (n = 656) (*Content*) (*Frequency*)
	CHAPS structured Initial Assessment with algorithm-identified CHAPS standard problems/topics in Microsoft Access	CHAPS Assessments (n = 140) and problems/topics identified (*Content*) (*Frequency*)
	Participant panel tracking tool in Microsoft Access	Tool cumbersome and discontinued; alternative secure methods used (*Content*)
Self-management Support		
	Prioritizing problems/topics with participant input	Most frequent concerns: • medications (*n* = 58 participants) • physical activity (n = 49 participants) • falls (n = 49 participants) (*Content*) (*Frequency*)
	Coaching by nurse care managers	• Education: verbal (*n* = 310), print (*n* = 168), Internet or DVD (n = 68) notes • Counseling and emotional support (*n* = 387 notes) • Motivational collaborative-problem solving (*n* = 144 notes) (*Content*) (*Frequency*)
	Siebens Health Care Notebook	• Participants (*n *= 83, 59.3%) interacted with Notebook • Problem-focused education sheets (*n* = 2.6 (SD 2.4)) added from 69 unique education sheets (*Content*) (*Frequency*)
	My HealtheVet (online tool for partnering with health care team with secure messaging)	Referral to My HealtheVet patient portal (n = 23 notes) (*Content*) (*Frequency*)

*CHAPS* Care Coordination for Health Promotion and Activities in Parkinson’s Disease

*APDA* American Parkinson Disease Association; LA-CRC – Los Angeles Caregiver Resource Center; *NPF* National Parkinson Foundation; PRO – Parkinson’s Resource Organization; Notebook – Siebens Health Care Notebook [18]

Note: Content, frequency, and duration per Hasson’s Model [21]

^a^ Of 140 participants who received the CHAPS Assessment, 119 received follow-up care management [14]

^b^ Siebens Domain Management Model™ has four domains: I Medical/Surgical Issues, II Mental Status/Emotions/Coping, III Physical Function, and IV Living Environment (© Hilary C Siebens MD 2005) [19]. Used with permission

^c^ One standard education sheet per domain added to each Notebook: I Levodopa and protein; II PD At Home (monthly education and support via telephone call); III Exercise and PD; IV Fall Proofing Your Home

^d^ Problem/Topic Intervention Protocol steps: Assess further, Provide information, Problem solve collaboratively, Clinical referrals, and Community and social services referrals [7, 14]

**Table 2:** CHAPS implementation fidelity moderators

Potential Moderator Types	CHAPS Implementation
Participant Responsiveness (i.e., individuals receiving and individuals delivering CHAPS)	◆ Patient/participants’ survey responses about CHAPS [20] • Positive (*n* = 246, 74%) • Neutral (*n* = 53, 16%) • Negative (n = 35, 10%) ◆ Patient/participants’ usability survey responses about the CHAPS Initial Assessment [20] • Positive (*n* = 51, 81%) • Neutral (*n* = 11, 17%) • Negative (n = 1, 2%) ◆ CHAPS nurse care manager survey responses noting specific program benefits [20] • Yes (*n* = 72, 74%) • Unsure (*n* = 19, 20%) • No (*n* = 6, 6%) ◆ Parkinson’s disease specialist survey responses noting specific program benefits [20] • Yes (*n* = 107, 77%) • Unsure (*n* = 30, 21%) • No (n = 3, 2%) ◆ CHAPS nurse care manager usability survey responses to Siebens Domain Management Model™ [20] • Facilitators in using the Model (*n* = 55, 65%) • Challenges in using a new Model (*n* = 29, 35%) ◆ CHAPS nurse care manager usability survey responses to the self-care Notebook [20] • Facilitators for coaching about the Notebook (*n* = 46, 62%) • Challenges to coaching about the Notebook (*n* = 28, 38%) ◆ Patient/participant reported feedback on Notebook to CHAPS nurse care managers [20] • Notebook assets (*n* = 97, 67%) • Notebook review deferred (n = 28,19%) • Reasons for not using Notebook (*n* = 19,13%) ◆ Principal Investigator observed CHAPS nurse care managers actively using CHAPS tools ◆ Neurology clinic physician assistant appreciated participants’ Notebooks
Comprehensiveness of policy description (i.e., CHAPS protocol)	◆ CHAPS intervention protocol published [7] ◆ Intervention implementation details published [14] ◆ CHAPS nurse care manager orientation (10–40 h) [14] ◆ Parkinson’s disease specialist & administrative staff orientation (1 h) [14] ◆ Content of CHAPS Nurse Care Manager Binder (7 sections) [14] ◆ Neurology leadership informed in person about CHAPS protocol (1 h) [14]
Strategies to facilitate implementation (i.e., supports for delivering CHAPS components)	◆ Print and online version of CHAPS Nurse Care Manager Binder [14] ◆ CHAPS nurse care manager hands-on practice of CHAPS Initial Assessment and Notebook during orientation [14] ◆ Principal Investigator (nurse researcher) was available and provided feedback on documentation and care management ◆ CHAPS nurse care manager conference calls twice monthly, then monthly – reported to Principal Investigator ◆ CHAPS nurse care manager huddles with Parkinson’s disease specialists monthly – reported to Principal Investigator [14]
Quality of delivery (i.e., extent to which provider (CHAPS) approaches theoretical ideals)	◆ Fidelity to Chronic Care Model achieved (Table 1) ◆ 5 patient-centered steps of the Nursing Process documented [14] ^a^ ◆ 5 intervention protocol steps to address problems/topics utilized [14] ^b^ ◆ Evidence-based Organizing Framework, Siebens Domain Management Model™ for holistic care management actively used [14] ◆ 140 (100%) of participants were provided 3-ringed binder self-care tool Notebook to encourage self-management [14] ◆ Nurse care managers discussed Notebook with participants (*n* = 108, 77%) ◆ CHAPS nurse care managers reported and documented participant self-care actions [14] • I Medically-focused (*n* = 239) • II Mentally/emotionally/coping-focused (*n* = 871) • III Functionally-focused (*n* = 196) • IV Environmentally-focused (*n* = 29) ◆ CHAPS nurse care manager and Parkinson’s disease specialist communicated regularly through huddles [14]
Recruitment including barriers to maintaining involvement of participants	◆ Recruitment performed through letters and telephone calls ◆ 140 of 162 (86%) of those randomized to intervention received nurse care management [17] ◆ 3 of these 140 (2%) declined after care management started [14]
Context (economic, organizational, community)	◆ Veterans Affairs Health Services Research and Development, Nursing Research Initiative funded the CHAPS trial ◆ Veterans Affairs open to quality of care improvement initiatives ◆ Veterans willing to participate in research ◆ Relationships with local community organizations (e.g., Parkinson’s support groups) ◆ Unable to incorporate CHAPS Initial Assessment and algorithms in electronic medical record (Computerized Personal Record System), requiring separate software ◆ Primary barrier to full intervention implementation was maintaining sufficient nurse care manager staffing due to Veterans Affairs hiring freeze in setting of normal turnover [14]

CHAPS – Care Coordination for Health Promotion and Activities in Parkinson’s Disease; Notebook – Siebens Health Care Notebook [18]

^a^ Nursing Process: Assessment, Nursing Diagnoses, Planning Outcomes, Implementing Interventions

^b^ Intervention Protocol Steps: Assess further, Provide information, Problem solve collaboratively, Clinical Referrals, Community and Social Service Referrals

A literature review of research articles published between 2012 and 2020 using search terms Parkinson’s disease, patient-centered care, and care coordination yielded articles enumerating challenges in Parkinson’s care delivery and research [6, 9–13, 28, 29]. Challenges likely to be addressed through nurse-led care management were identified.

### Analyses

Open card sorting methodology was used to organize Parkinson’s care challenges identified in the literature. Two researchers (KIC, HCS) together examined care challenges for word similarities (generalizations in semantics, analogies, and metaphors). They sorted these challenges into groups that were not pre-specified [30, 31]. They used their knowledge of healthcare and language to refine the sorts. For items on which they disagreed, they came to a collaborative decision for placement in a group. These groups were given names and all data (themes and associated items) were entered into a table (Table 3). Then these researchers examined CHAPS nurse care manager activities [14] and stakeholder perception survey responses [20] for examples addressing each challenge item and these were added to Table 3.

**Table 3:** Challenges in Parkinson’s disease care management design addressed in the CHAPS Model

Challenges	Examples of CHAPS Addressing the Challenges ◆ Quality and Extent of Implementation [14] ○ Stakeholder Perceptions [20]
THEME 1: UNMET NEEDS (IDENTIFIED BY PATIENT AND/OR CARE PARTNER)	
Lack of emotional support [28, 32]	◆ Emotional support through CHAPS nurse care manager coaching ◆ Participants attended support groups
	○ Participants felt they could talk to their nurse care manager
Need for tailored information [28, 32, 33]	◆ CHAPS nurse care managers: • Provided CHAPS Assessment-driven education (verbal, written, digital) • Recommended specific care interventions • Personalized participant Notebook [18] with tailored education sheets ◆ Participants read specific nurse care manager-supplied materials
	○ Participants liked the Notebook feature of education sheets
Coping with multiple changes in care (unpaid care needs, medications, adaptive equipment) [33]	◆ Motivational collaborative problem-solving ◆ CHAPS nurse care manager coaching
	○ Participants reported CHAPS nurse care managers helped them manage their Parkinson’s disease and their health overall
More self-management [33, 34]	◆ CHAPS nurse care managers coached participants on self-care including My HealtheVet and Notebook use ◆ Participant self-care actions and interactions with Notebook were documented
	○ Participants reported: • Medication self-management • Knowing about Parkinson’s disease and when to contact Parkinson’s disease specialists • Notebook benefits (helpful, useful, organizes information) ○ Parkinson’s disease specialists reported participant self-management improvement
More active role in decision-making [35]	◆ Participants prioritized problems with CHAPS nurse care manager ◆ Collaborative problem-solving occurred ◆ Participants prepared for provider appointments
	○ Participants felt they could talk to their nurse care manager
More time to discuss the future, possible scenarios [33, 34]	◆ CHAPS nurse care managers: • Made follow up telephone calls to participants • Discussed Understanding Parkinson’s Disease, Preferences/Long term care planning, End of Life Resources ^a^ • Added education sheets on above issues to personalized Notebooks ◆ Participants completed advance directive/power of attorney for health care
	○ Parkinson’s disease specialist appreciated nurse care manager spending more time talking to patients than is available in clinic
THEME 2: SUGGESTIONS FOR PROVIDERS (IDENTIFIED BY PATIENT AND/OR CARE PARTNER)	
Health professional as single point of access for problem-solving directly or for multidisciplinary care and referrals [10, 34]	◆ CHAPS nurse care managers: • Problem-solved collaboratively with participants • Initiated care coordination and discussed multidisciplinary referrals • Recommended topic/intervention to discuss with provider
	○ Parkinson’s disease specialists noted helpfulness of the nurse care manager role
Continuity of contact needed [28, 32]	◆ Continuity achieved for some but not all participants; continuity interrupted by normal turnover complicated by hiring freezes
	○ CHAPS nurse care managers noted consistent staffing is needed to build trust, facilitate collaboration, foster behavioral change, and support Notebook use
Better interdisciplinary collaboration [34]	◆Facilitation of interdisciplinary communication using the Siebens Domain Management Model ^b^ ◆ Care coordination through warm hand-offs ◆Monthly clinical huddles between nurse care managers and Parkinson’s disease specialists ◆ Participants taking Notebook to provider appointments
	○ Parkinson’s disease specialists reported CHAPS nurse care managers recommended care suggestions they agreed with (e.g., in clinical huddles, in documentation)
Competent, professional practice [32]	◆Structured CHAPS Assessment with algorithms (embedded triggers) for problem/topic identification ◆ Scheduled follow-up telephone calls for follow-through and proactive care ◆ Problem/topic specific intervention protocols ^c^
	○ CHAPS nurse care managers gained knowledge/understanding about Parkinson’s disease ○ Parkinson’s disease specialists reported CHAPS nurse care managers provided relevant information and paid attention to detail
THEME 3: PATIENT CHARACTERISTICS NEEDING CONSIDERATION	
Variability of disease severity in Parkinson’s disease [6, 36, 37]	◆ CHAPS Assessment with embedded triggers for identification of problems/topics and their range of severity (e.g., physician referral for higher severity) ◆ Problems/topics spanning early, mid to advanced Parkinson’s disease (e.g., Driving, Psychosis/Hallucinations ^a^)
	○ CHAPS nurse care managers agreed with care suggestions recommended by CHAPS Assessment (triggered by algorithms)
As disease progresses, anticipation of needs is required [33]	◆ Proactive telephone calls over time ◆ CHAPS 6-month review and annual reassessments to screen for evolving problems
	○ Participants aware of what Parkinson’s disease symptoms to watch for
THEME 4: STANDARDIZING MODELS FOR PARKINSON’S CARE MANAGEMENT	
Standardized models for Parkinson’s team-based care are needed [6, 9, 11–13, 29, 38]	◆ CHAPS Assessment with algorithms ◆ Siebens Domain Management Model ^b^ ◆ Participants prioritized problems with CHAPS nurse care manager ◆ Problem/topic-specific intervention protocols ^c^ ◆ Monthly clinical huddles of CHAPS nurse care managers and Parkinson’s disease specialists ◆ My HealtheVet and Notebooks for self-care and team communication ◆ Care partner included at participant’s request ◆ Referrals to and collaboration with other disciplines ◆ Communication through one shared electronic medical record
	○ Overall stakeholder perceptions of CHAPS and its components were positive ○ Parkinson’s disease specialists and nurse care managers endorsed CHAPS (e.g., would refer other patients)
Importance of including patient perspective in team [6, 10]	◆CHAPS nurse care manager elicited participant concerns about CHAPS problems/topics and other medical problems
	○ Participants felt they could talk to the nurse care manager about their condition ○ Participant preferences guided Notebook coaching
Care partner stress to be considered [6] and care partner included in team [10, 11]	◆ Care partners participated in telephone calls and care coordination at participant request ◆ Participants showed Notebook to care partner ◆ Caregiver Packets sent ^d^
	○ Care partner responses to the Notebook included being impressed and reporting it was helpful/organized

CHAPS – Care Coordination for Health Promotion and Activities in Parkinson’s Disease; Notebook – Siebens Health Care Notebook [18]

^a^ These are among the 31 CHAPS standard problems/topics

^b^ The Siebens Domain Management Model™ is an organizing framework with four domains: I Medical/Surgical Issues, II Mental Status/Emotions/Coping, III Physical Function, and IV Living Environment (© Hilary C Siebens MD 2005) [19]. Used with permission

^c^ Problem/Topic Intervention Protocol steps were: Assess further, Provide information, Problem solve collaboratively, Clinical referrals, and Community and social services referrals

^d^ Caregiver Packet included a self-administered Caregiver Strain questionnaire, a screen for Caregiver Mood (Personal Health Questionnaire-9), a resource list, and caregiver information sheets from Parkinson’s Disease Foundation

## Results

### Fidelity to the chronic care model

Table 1 lists CHAPS components, in column 2, for each of the six Chronic Care Model elements; thus, the CHAPS Model achieved fidelity to this theoretical model. For the elements of Delivery System Redesign, Decision Support, and Self-management Support, there were multiple components for supporting CHAPS nurse care managers, Parkinson’s disease specialists, and participants.

### Fidelity to CHAPS implementation

#### Adherence

CHAPS Model components, listed in Table 1, were implemented as demonstrated by *content*, *frequency*, and *duration* results (column 3 of Table 1), achieving full implementation fidelity for all components except for partial fidelity to telephone encounter frequency. Adherence *coverage,* as defined by the Hasson model, was the proportion of a target group that received the intervention [21]. For CHAPS, this was defined *post-hoc* as, at minimum, receipt of the CHAPS Initial Assessment. Adherence *coverage* was 86% (*n* = 140 of the 162 intervention arm participants).

#### Potential moderators

Potential moderators during the CHAPS implementation were identified (Table 2) for each of the six conceptional framework categories [21] (Fig. 1). First, concerning *participant responsiveness,* survey results at study end indicated engagement by all three stakeholder groups (i.e., patient/participants, CHAPS nurse care managers, and Parkinson’s disease specialists). Their feedback was overall positive [20]. CHAPS nurse care manager usability responses included facilitators for both the Siebens Domain Management Model and the Notebook and the three attributes: user-friendly, person/patient-centered, and organized. Second, *comprehensiveness of policy (protocol) description* consisted of already published data describing the study protocol [7] and details of the CHAPS implementation [14]. Third, *strategies to facilitate implementation* included educational materials, practice with CHAPS tools, and scheduled communications for decision support. Fourth, *quality of delivery* modes included multiple care management components for proactive holistic Parkinson’s care and nurse care managers’ attention to participants’ self-care actions [14]. Fifth, *recruitment* and maintaining participant involvement was effective [14]. Sixth, some aspects of the CHAPS implementation’s *context* provided support for the randomized controlled trial while other aspects were barriers to full implementation. For example, a hiring freeze resulted in CHAPS nurse care manager availability for only 68% median CHAPS nurse care manager days of coverage, interquartile range, 47–100% [17].

#### Control group

In examining implementation fidelity, Hasson recommended a description of the control group. In the CHAPS parent study, both intervention and control (usual care) groups had access to Veterans Affairs PADRECCs present at all five sites to provide medical, educational, and support services to patients with movement disorders [39]. All participants received a brief educational handout on Parkinson’s [40] and had similar numbers of patient/participant interactions by visit and provider types from baseline to 18 months [17].

The control group (usual care) received no CHAPS Initial Assessments. Of note, they were assessed significantly less frequently for excessive daytime sleepiness (14% vs 85%, *p* < 0.0001) and orthostatic hypotension (41% vs 86%, p < 0.0001), psychosis/hallucinations/delirium (60% vs 94%, p < 0.0001), cognition (70% vs 93%, p < 0.0001), depression (80% vs 94%, *p* = 0.0002), and falls (74% vs 96%, p < 0.0001) [17]. These participants did not receive the CHAPS self-care Notebook and they received significantly fewer telephone calls (0.11 SD 0.2 versus 3.02 SD 2.2).

### Parkinson’s care challenges from the literature and the CHAPS implementation

Four themes emerged for grouping related Parkinson’s care challenges through open card sorting after three iterations: (1) Unmet Needs Identified by Patient and/or Care Partner (six items) [28, 32–35], (2) Suggestions for Providers Identified by Patient and/or Care Partner (four items) [10, 28, 32, 34], (3) Patient Characteristics Needing Consideration (two items) [6, 33, 36, 37], and (4) Standardizing Models for Parkinson’s Care Management (three items) [6, 9–13, 29, 38] (Table 3). These challenges were addressed in the CHAPS implementation through a defined CHAPS nurse care manager role and standardized CHAPS components. These nurse care managers tailored activities using problem/topic intervention protocol steps: (1) assess further, (2) provide information, (3) problem solve collaboratively, (4) clinical referrals, and (5) community and social services referrals [7, 14]. We also identified examples of stakeholder perceptions related to the challenges (Table 3) [20].

## Discussion

The CHAPS Model and its multiple components reflected all six essential elements of the Chronic Care Model, thus achieving fidelity to this model. The CHAPS Model components were delivered as intended, thus achieving full implementation fidelity. This was demonstrated by adherence to content, frequency, and duration except for partial fidelity to telephone encounter frequency. We identified potential fidelity moderators for all six of Hasson’s moderator types. Stakeholders’ (participants, CHAPS nurse care managers, Parkinson’s disease specialists) responsiveness indicated CHAPS relevancy to patient care. Additionally, the CHAPS implementation addressed recently cited challenges in Parkinson’s care management.

### Limitations

The subgroup studied (*n* = 140) was mostly male, all veterans, and they were receiving care within the Veterans Affairs Medical Centers. These characteristics may limit generalizability of findings in future implementations. However, over half of the participants received care from other health systems like other individuals with Parkinson’s who are receiving care from multiple sources. The literature abstraction of challenges in Parkinson’s care was not exhaustive and additional challenges may be identified by others.

The association of CHAPS components to the clinical outcome of fewer depressive symptoms on a depression screener [17] was not analyzed as is recommended for evaluating fidelity (Fig. 1). The importance of this type of analysis was demonstrated in the ACCESS study when one of many activities, a home assessment, was associated with improved caregiver mastery at 18 months [41]. Future CHAPS implementations could assess component and clinical outcome relationships.

### Implications

#### Simplification of care delivery

Given the complexity of Parkinson’s care, a strength of CHAPS is the simplification of care delivery through discrete, defined, and transparent structured components for reproduceable dissemination. Quality of delivery was evidenced by meeting PD quality of care indicators and garnering positive stakeholder feedback.

#### Organized and relevant documentation

The 4-domain Siebens Domain Management Model, as the Organizing Framework, applied in documentation, allows clinicians to find information about areas of greatest concern. For example, if pneumonia prevention is a concern for Parkinson’s disease specialists or primary care providers, they can review Domain I to learn about a patient’s swallowing status. If psychiatrists are concerned about hallucinations or delirium, they can focus on acute medical issues in Domain I that could be contributory to cognitive issues documented under Domain II. Any provider with concerns about function (Domain III) or the home situation (Domain IV) can quickly review these sections.

The CHAPS structured documentation by CHAPS nurse care managers may initially appear as a documentation burden, a known contributor to clinician burnout [38]; however, these nurse care managers obtained and documented information that both they and Parkinson’s disease specialists appreciated and found relevant [20]. For example, motor and non-motor signs and symptoms were assessed, and managed, significantly more frequently than in the usual care group [17]. The process of assessing Parkinson’s disease signs and symptoms - per the PD quality indicators - are not clinical or patient-reported outcomes [38], yet we believe they are necessary precursors to managing these problems if participants’ outcomes are to be improved.

#### Comparisons with nursing roles in other Parkinson’s models of care

CHAPS addresses nursing assessments, care coordination, and care management similar to published information on the Dutch nursing guidelines in ParkinsonNet [14, 42, 43] and the Integrated Parkinson’s Care Network [44, 45]. All three programs utilize nurses with specialized knowledge in Parkinson’s and address self-management support needs of individuals with Parkinson’s. All have a network approach. CHAPS was administered in one of 6 regional Veterans Affairs Centers of Excellence for Parkinson’s disease, called PADRECCs, in the United States with building blocks for networked care [46]. The PADRECCs expanded care of Parkinson’s through a national Veterans Affairs consortium of providers with movement disorder expertise.

ParkinsonNet proposes 1:370 full-time employee equivalent (FTEE) nurse to individual with Parkinson’s with additional help from a care coordinator, not necessarily a nurse [42]. CHAPS achieved a ratio of 1:125 FTEE nurse care manager to individuals with Parkinson’s without any coordinator assistance. CHAPS contact time and frequency [17] are like those proposed for ParkinsonNet nurses [43].

To the best of our knowledge, the proactive CHAPS intervention is the first to document improved Parkinson’s care quality through nurse care managers oriented to Parkinson’s using a set of specific tools. These tools included (1) a standardized CHAPS Assessment regardless of disease stage, with algorithms to trigger problems/topics and their severity for care management, (2) an evidence-based, interdisciplinary person/patient-centered Organizing Framework (the Siebens Domain Management Model), (3) 5-step problem/topic intervention protocols for decision support for nurse care managers, (4) a personalized self-care plan, “My Action Plan”, with coaching by the CHAPS nurse care managers, and (5) an individualized self-care Notebook with teaching in its use. The overall contact frequency approach was flexible with contacts every 6 months and more frequently if needed.

#### Contributing insights to care management of enduring health conditions

CHAPS, as a care management program, includes communication, collaboration, and coordination like other successful non-Parkinson’s care delivery studies. The Advanced Illness Coordinated Care Program [47] provided health counseling, education, and care coordination in a 6-session intervention focused on individuals’ disease status and long-term planning. This intervention was based on a 3-domain biopsychosocial model. However, CHAPS used the 4-domain Siebens Domain Management Model [19], including function, which is consistent with the biopsycho-ecological model. CHAPS nurse care managers assessed participants’ understanding of their Parkinson’s and planning for future needs (e.g., discussion about surrogate decision-maker, which occurred more frequently than in usual care group [17]). In collaborative care for patients with depression and chronic illness [48], nurses received decision-support from physicians, worked collaboratively with them, and followed guideline-based protocols, as occurred similarly in CHAPS. In the Medicare Coordinated Care Demonstration [49], six activities were identified across four programs that reduced hospitalizations in high-risk patients. These activities occurred in CHAPS through nurse care managers who: (1) were a single point of contact, (2) met regularly with Parkinson’s disease specialists, (3) delivered evidence-based education to patients, (4) provided tailored medication management, and (5) addressed transitional care (e.g., medication review, discharge instructions) via telephone. Finally, (6) in-person meetings to supplement telephone calls were used a few times in CHAPS (*n* = 23 (3.5%) of 656 notes) [14]. Given these care management components common to prior studies and CHAPS, health systems and providers may wish to examine their Parkinson’s care management delivery and determine if they can use the CHAPS Model, and its components, building on their current strengths.

#### Importance of stakeholder feedback in research

The Care and Learn model for improving care quality in health care delivery highlights the importance of people involved (patients and clinicians) and a holistic approach to patients and their context. The model advocates for gaining evidence about the caring provided and on stakeholder learning including program acceptability [50]. CHAPS followed these considerations. The caring provided was described in detail using documentation review [14]. The focus on individuals is reflected in the CHAPS stakeholder feedback. Patients reported helpfulness of the CHAPS nurse care manager, acceptability of the CHAPS Initial Assessment, overall knowledge about Parkinson’s disease care, and Notebook benefits and features liked. CHAPS nurse care manager and Parkinson’s disease specialist surveys addressed knowledge/understanding, self-confidence, clinical appropriateness, participant’s self-management improvement, and program endorsement. Overall, these surveys provided positive feedback and some suggestions for refinements. Additionally, surveys on usability of new tools (i.e., Siebens Domain Management Model, CHAPS Assessment, and Siebens Health Care Notebook) provided evidence of their acceptability [20].

#### Telehealth

The literature supports providing Parkinson’s care remotely (telehealth via telephone and/or home video or clinical video telehealth) in both rural and urban areas. Telehealth is advantageous when access to specialists is limited, patients have trouble keeping appointments, specialists have inadequate time per patient, and coronavirus disease (COVID 19) precludes some in-person visits [51–55]. These findings add support to CHAPS as a telephone-based program. In the future, CHAPS could be augmented with clinical video telehealth when clarity in assessment or face-to-face teaching are needed. Other telehealth applications could include zoom/virtual classes for individuals’ education and support (e.g., introductory class to the self-care Notebook tool) by CHAPS nurse care managers organized by support staff. Future digital technologies like wearable sensors (e.g., detection of sleep disturbances, on/off phenomena) and their data could also be integrated into proactive CHAPS care management [56].

#### Safety

Among the multiple dimensions of safety [57, 58], CHAPS addressed four directly: *care integration*, *patient engagement*, *meaningful work for providers*, and *broader interdisciplinary approaches* [59–61]. First, *care integration* started with CHAPS nurse care managers proactively identifying participants’ safety risks through a structured CHAPS Assessment with embedded algorithms. Assessment Summary reports were shared through electronic medical record documentation, available to inpatient and outpatient Veterans Affairs providers, and through paper copies in participants’ personalized Notebooks. Structured team communications were designed for care integration through collaboration and coordination (e.g., clinical huddles, orderly documentation format/template using the Siebens Domain Management Model). The self-care Notebook served as a care integration tool, portable to any venue (inpatient, outpatient, residential), especially important for urgent care and emergencies [62]. Second, CHAPS fostered *patient/participant engagement* through nurse care manager coaching in specific actions and, at times, including the care partner (e.g., when to call Parkinson’s disease specialist, using electronic and/or hard copy self-care tools based on participants’ preferences [14, 20, 63, 64]). Third, the CHAPS Program facilitated *meaningful work* for the nurse care managers and Parkinson’s disease specialists (e.g., CHAPS Assessment provided information that would improve their patient care, their patients had a better understanding of how to manage their Parkinson’s). Furthermore, nurse care managers and Parkinson’s disease specialists endorsed CHAPS (81% would refer other patients to CHAPS) [20]. Fourth, *broad interdisciplinary approaches* occurred in CHAPS through referrals (*n* = 501) to providers, Veterans Affairs services, and community services. Warm hand-offs (*n* = 358) between nurse care managers and other health care professionals occurred frequently through live telephone discussions, concurrent co-signatures on specific notes, secure email, and face-to-face discussions [14].

## Conclusion

CHAPS, a supportive nurse-led proactive Parkinson’s care management program, improved care quality and is designed to be reproducible and supportive to clinicians. Findings indicated CHAPS Model fidelity occurred to the Chronic Care Model and to implementation of the CHAPS components. Current Parkinson’s care management challenges were met through CHAPS activities. Thus, dissemination of CHAPS merits consideration by those responsible for implementing changes in clinical practice and reaching people in need.

## Data Availability

The datasets generated and/or analyzed during the current study are not publicly available as, by the time we deidentify data to the degree acceptable, too many key variables are taken out given that veterans can be re-identified with enough social and/or personal demographic and area information. Additionally, the small pool of intervention nurse care managers and Parkinson’s disease specialists would potentially allow tracing qualitative survey data back to them. The data that support the findings of this study are from the Veterans Affairs, USA; thus, restrictions apply to the availability of these data. Data are however available upon reasonable request from and with permission from the Center for the Study of Healthcare Innovation, Implementation and Policy, Veterans Affairs, Los Angeles, California, USA.

## Abbreviations

CHAPS: Care Coordination for Health Promotion and Activities in Parkinson’s Disease
PD: Parkinson’s disease
ACCESS: Alzheimer’s Disease Coordinated Care for San Diego Seniors
ACOVE: Assessing Care of Vulnerable Elders
PADRECC: Parkinson’s Disease Research, Education and Clinical Centers
ICD 9: International Classification of Disease − 9
Notebook: Siebens Health Care Notebook
SD: Standard deviation
FTEE: Full-time employee equivalent
COVID 19: Coronavirus disease
VA HSR&D-NRI: Veteran Affairs Health Services Research and Development – Nursing Research Initiative
USA: United States of America

## Footnotes

1 Terminology is evolving for the name “Parkinson’s disease”. We refer to it as Parkinson’s, de-emphasizing the “disease” label in this paper except when it is a proper name in a title, as in Care Coordination and Health Activities in Parkinson’s Disease (CHAPS), the original intervention’s title and in referring to Parkinson’s disease specialists. As CHAPS is disseminated after this implementation, its title will be Care Management and Health Activities in Parkinson’s as care coordination is only one component of the comprehensive care management provided by this intervention.
